# 

**Published:** 1808-04

**Authors:** 


### NEW MEDICAL PUBLICATIONS.

Remarks on the Purulent Ophthalmy, which has lately been epidemical in this country; by James Ware, Surgeon, F.R.S. 2s. sewed.

A Practical Treatise on the Radix Rhataniæ, or Rhatany Root; to which are added, Directions for the Use of the Phosphate and Oxyphosphate of Iron in Cancerous Affections; by Richard Reece, M.D. 2s.

A Letter to Sir Joseph Banks, Bart. F.R.S. on the Causes and Removal of the prevailing Discontents, Imperfections, and Abuses in Medicine; by Thomas Beddoes, M.D. 4s.

The Muscular Motions of the Human Body; by John Barclay, M.D. Lecturer on Anatomy. 8vo. 12s.

The Principles of Surgery; by John Bell, Surgeon. Vol. 3, Royal 4to. 2l. 2s.

